# Assessment of the knowledge, attitude and practices of the informed consent process in oral healthcare among dental students in Makerere University Dental Hospital, Uganda

**DOI:** 10.1186/s12909-024-05520-0

**Published:** 2024-05-10

**Authors:** David Nono, Ernest Mwebesa, Godfrey Bagenda, Isaac Okullo, Charles Mugisha Rwenyonyi, Simon Williams

**Affiliations:** 1 Faculty of Life Sciences and Education, University of South Wales, Cardiff, UK; 2 Learna Ltd Ty Bevan, House Cleeve Drive LIanishen, Cardiff, CF14 5GF UK; 3 School of Dentistry, College of Health Sciences, Makerere University, Kampala, Uganda; 4 School of Biomedical Sciences, College of Health Sciences, Makerere University, Kampala, Uganda

**Keywords:** Informed consent process, Knowledge, Attitudes, Clinical practices, Dental students

## Abstract

**Introduction:**

Informed consent is an ethical and legal component of healthcare. It ensures patient autonomy and allows patients to make decisions regarding their treatment. In dental care, informed consent is particularly important because most dental procedures are invasive. Since dental students are future dentists, they need to learn about their ethical obligations and accountability through the informed consent process as this is critical to patients’ well-being. This study aimed to determine dental students’ knowledge, attitudes, and practices of the informed consent process for oral health care in Makerere University Dental Hospital, Uganda.

**Study methodology:**

This was a descriptive cross-sectional study using quantitative methods. It was carried out at Makerere University Dental Hospital and third, fourth, and fifth-year students (*n* = 102) pursuing a Bachelor of Dental Surgery program took part in the survey. A self-administered structured questionnaire was used to assess their knowledge, attitudes, and practices of informed consent for oral health care. Collected data were entered into Epi-data version 3.1, where it was cleaned, coded, and imported to STATA version 14 software for statistical analysis.

**Results:**

About two-thirds 67 (65.7%) of the participants were males. The mean age was 25 (SD = 3.21) years. The majority (90%) of the students had a high level of knowledge of the informed consent process. About (80%) had a positive attitude towards informed consent and (85%) most often practiced the informed consent process. Based on bi-variate analysis, training on informed consent, year of study, age, and sex were significantly associated with the informed consent process. However, there was no significant risk factor associated with informed consent in multiple logistic regression analysis.

**Conclusion:**

The study findings highlighted high levels of knowledge, positive attitude, and practice of the informed consent process among the clinical dental students. Continuous training is necessary to remind dental students about the importance of informed consent in healthcare, not only for complex procedures.

## Background

Informed consent is a constitutive/essential component of the healthcare profession [1]. It is also an important legal and ethical principle, rooted in the patients’ autonomy [2, 3]. Patients have a right to make an informed decision (in other words, they should decide on treatment after understanding the condition they have, and available treatment options, including risks and benefits associated with each option) related to their care or treatment without any form of coercion [2]. They have the freedom to choose to consent to or decline treatment as opposed to relying on the decision of the health care providers [1].

The requirement to obtain informed consent from patients before providing medical or dental care should be common practice [4–5] before any procedure due to related costs with unforeseen attendant complications or risks [6, 7]. During dental training, students need to learn about their ethical obligations and accountability regarding their patients as this is critical to the patient’s well-being [8]. In dental care, some procedures are invasive, especially surgical procedures, hence it is required for dental practitioners to seek written informed consent from patients before such procedures are carried out [9]. In some instances, dentists do not seek informed consent or in most cases, they obtain verbal consent [10]. This can cause dissatisfaction and mistrust among patients towards dental practitioners hence can lead to medico-litigation. In addition, Article 10 of the Uganda Medical and Dental Practitioners Council Code of Professional Ethics states that consent may be given verbally, however, it would be ideal to have the consent documented to protect medical practitioners against possible litigation in some circumstances. The documented consent can serve as proof that the patient was informed about the possible benefits and risks of the treatment they were going to receive [10].

In a study done at Mulago National Referral Hospital among dental practitioners, Nono et al. [10] found that only 5.3% obtained written informed consent from patients. In addition, in a study done at Uganda Cancer Institute among cancer healthcare professionals, Kampi et al. [11] found that most of the respondents were dissatisfied with the way informed consent was documented and that patients just give verbal consent for medical procedures, some of which are complex and invasive. Dental students are future practitioners, hence they are expected to incorporate the informed consent process in their dental practice and this should start early during their clinical training. Positive attitudes towards informed consent promote patient-centered care and contribute to improved patient satisfaction and trust in the dental profession. On the other hand, negative attitudes about informed consent can hamper effective communication and compromise patient care.

However, there was no known published study on dental students’ knowledge, attitudes, and practices of obtaining informed consent for oral health care in Uganda. There was a need to ascertain how knowledgeable dental students are, their attitudes, and their practices of obtaining informed consent from patients as they pursue their studies. Therefore, this study aimed to establish dental students’ knowledge, attitudes, and practices of obtaining informed consent for oral health care of patients in Makerere University Dental Hospital, Uganda.

## Materials and methods

### Study design

This was a descriptive cross-sectional study that collected data through a survey using a questionnaire.

### Study site

The study was conducted at Makerere University Dental Hospital in Kampala, Uganda. Kampala is the capital city of Uganda, and it is politically designated as a district. The hospital is a teaching and health service delivery facility of Makerere University. The site was chosen because it is the largest dental hospital and has the highest number of dental students in Uganda. It offers specialized dental care to staff, students, and other patients from outside the University at a minimal fee, making it easier to attract more patients compared to other nearby facilities. The dental hospital attends to approximately 660 outpatients per month (Registry of Dental Records, 2022). Bachelor of Dental Surgery is a 5-year program at Makerere University. The dental surgery students become clinical students in their 3rd year.

### Selection of study participants

The study participants were clinical (3rd, 4^th,^ and 5th ) year dental students pursuing a Bachelor in Dental Surgery (BDS) program and who provided written informed consent to participate in the study. All the students in the 3rd, 4^th,^ and 5th year were 102 in total and all were selected for the survey using a total population sampling technique because the total population was very small and had a particular set of characteristics of interest for the study.

### Inclusion criteria

Third, fourth, and fifth-year dental students pursuing a degree program in Dentistry at Makerere University and were willing to provide written informed consent to participate in the study.

### Exclusion criteria

Dental students who were absent or not available during the data collection period.

### Data collection procedure

Participants were invited to a large room at the Dental Hospital to explain to them the purpose of the study. Written informed consent was obtained from each willing participant before data collection. The participants (*n* = 102) were given a self-administered questionnaire with close-ended questions to fill. The questionnaire comprised sections on socio-demography, knowledge, attitudes, and practices of obtaining informed consent in oral health care [12–15]. The statements on knowledge and attitudes were based on the Likert scale: “agree”, “disagree”, or “unsure” about the statements relating to the informed consent process.

For practices, respondents were asked if they fulfilled some aspects of informed consent processes either “always”, “most times”, “sometimes”, “rarely”, or “never”.

### Data management and analysis

The collected data were entered into Epidata version 3.1 software, cleaned, and double-checked for errors and completeness. Data were exported to STATA version 14 software for analysis. Descriptive statistics were used to summarize the data in frequencies and proportions and the results were presented in tables. Cross-tabulations between the dependent and independent variables were used to determine the association between the variables. The inferential analysis was employed using multiple logistic regression to generate odds ratios with their 95% confidence intervals to determine the strength of the association between the dependent and independent variables. *P*-value < 0.05 was considered statistically significant.

### Ethical consideration

Ethical approval of the study protocol was obtained from Mulago Hospital Research and Ethics Committee (Reference Number: MHREC-2544) as well as the University of South Wales, Faculty of Life Sciences and Education Ethics Group (Reference Number: 270,701 h). Permission to carry out the study was obtained from the administration of Makerere University Dental Hospital. Written informed consent was obtained from the participants in accordance with the Helsinki Declaration [16]. Confidentiality was observed by assigning each participant a unique identification number that was used on the study questionnaire and only known by the investigator. All collected data were kept securely under lock and key and only accessible to the investigator.

## Results

A total of 102 clinical dental students participated in the study and of these, 67 (65.7%) were males (Table 1). The mean age was 25 (SD = 3.2) years. The majority of the students 47 (46.1%) were in their 5th year of study. Most 76 (74.5%) of the respondents had received training in the informed consent process and most of the training was in the form of lectures (Table 1).

**Table 1:** Frequency distribution of the respondents according to socio-demographic characteristics

Characteristics of respondents	Frequency (*n*) *N* = 102	Percentage (%)
Age (years)	Mean Age = 25.0	Standard Dev = 3.2
**Sex**		
Male	67	65.7
Female	35	34.3
**Year of Study**		
3rd	25	24.5
4th	30	29.4
5th	47	46.1
**Training on informed consent**		
Yes	76	74.5
No	26	25.5
**If yes, in which form?**		
Lectures	71	93.4
Others	5	6.6
Total	102	100

Participants’ knowledge of the informed consent was based on a Likert scale. The respondents who had correct answers for each statement were considered as “agree” which were then constituted into a composite variable as a “high” level of knowledge while those who had incorrect answers were considered as “disagree” or “unsure” and were considered as “low” level of knowledge. At least 99 out of 102 respondents had high knowledge of the informed consent process (Table 2). There were a few instances in which the respondents expressed limited knowledge like seeking consent only for complex dental procedures 8 (7.8%) and whether patients should give consent under the influence of medication or alcohol 13 (12.7%) or no consent required for children 24 (23.5%) (Table 2).

**Table 2:** Frequency distribution of the respondents according to their knowledge of obtaining informed consent for oral health care

**Statement**	Knowledge Level	
	High n (%)	Low n (%)
It is the patient’s right to be informed about the treatment.	99 (98.0)	3 (2.0)
A patient has the right to accept or refuse treatment.	98 (96.1)	4 (3.9)
Informed consent is a legal requirement for dental procedures.	93 (91.2)	9 (8.8)
Informed consent is only necessary for complex dental procedures.	8 (7.8)	94 (92.2)
Informed consent is an ongoing process, not obtained once.	83 (81.4)	19 (18.6)
Informed consent can be obtained verbally or in writing.	80 (78.4)	22 (21.6)
A patient can provide informed consent if they are under the influence of medication or alcohol.	13 (12.7)	89 (87.3)
Informed consent is only necessary for adult and not pediatric patients.	24 (23.5)	78 (76.5)
A patient can withdraw his/her informed consent at any time during dental care.	84 (82.4)	18 (17.6)
It is necessary to inform a patient of all possible risks or complications associated with a dental procedure.	95 (93.1)	7 (6.9)
A patient can take legal action if not properly informed about risks or complications.	92 (90.2)	10 (9.8)

Respondents’ attitude toward the informed consent process was based on a Likert scale where those who indicated “agree” were constituted into a composite variable as having a “positive” attitude while those who selected “disagree” or “unsure” were considered as having a “negative” attitude. Dental students’ attitude towards the informed consent process was generally positive except for a few responses where 29 (28.4%) of the respondents were unsure or did not agree (negative attitude) that “obtaining informed consent can lead to better patient outcomes or satisfaction” and 24 (23.5%) of them did not agree or were unsure that “obtaining informed consent can help build trust and improve communication with patients” (Table 3).

**Table 3:** Frequency distribution of the respondents according to their attitude of informed consent for oral health care

Statement	Attitude	
	Positive n (%)	Negative n (%)
Obtaining informed consent is an important part of dental care.	94 (92.2)	8 (7.8)
Obtaining informed consent can help protect the patient’s autonomy or right to make decisions about their healthcare.	79 (77.5)	23 (22.5)
Obtaining informed consent can lead to better patient outcomes or satisfaction.	29 (28.4)	73 (71.6)
Patients have the right to refuse a dental procedure even if it is recommended by the dentist.	95 (93.1)	7 (6.9)
Obtaining informed consent is only necessary for high-risk dental procedures.	83 (81.4)	19 (18.6)
My dental education or training has adequately prepared me to handle the informed consent process.	92 (90.2)	10 (9.8)
Obtaining informed consent can help reduce the risk of litigation against a dentist.	91 (89.2)	11 (10.8)
Dental students should receive more training on the informed consent process.	86 (84.3)	16 (15.7)
Obtaining informed consent can help build trust or improve communication with patients.	24 (23.5)	78 (76.5)
I feel confident in my ability to explain the risks or benefits of dental procedures to patients.	75 (73.5)	27 (26.5)
Obtaining informed consent from patients is time-consuming and affects a dentist’s productivity.	93 (91.2)	9 (8.8)
Patients are more likely to comply with recommended dental procedures if they have been involved in the informed consent process.	91 (89.2)	11 (10.8)

Participants’ clinical practices on informed consent were based on a Likert scale. The respondents who had indicated “always” and “most times” were constituted into a composite variable as “often” while those who responded as “sometimes” “rarely” or “never” were considered as “less often”. The dental students’ clinical practices regarding the informed consent process for oral health care were generally high (Table 4).

**Table 4:** Frequency distribution of respondents according to clinical practices on informed consent for oral health care

Questions	Clinical practices	
	Often n (%)	Less often n (%)
Do you inform patients about their medical condition(s) or the procedures of the treatment?	95 (94.06)	7 (5.94)
Do you explain the risks or benefits of the proposed dental treatment to the patient before obtaining informed consent?	88 (87.13)	14 (12.87)
Do you discuss alternative treatment options with the patient before obtaining informed consent?	87 (86.14)	15 (13.86)
Do you inform the patient about possible consequences if he/she refuses the treatment?	90 (89.11)	12 (10.89)
Do you ensure that the patient has adequate time to ask questions before obtaining informed consent?	99 (98.02)	3 (1.98)
Do you obtain written informed consent from the patient before starting the dental procedure?	91 (90.10)	11 (9.90)
Do you obtain informed consent from the patient’s legal guardian or next of kin if the patient is unable to give consent?	77 (76.24)	25 (23.76)
Do you inform the patient about the costs associated with the proposed dental treatment before obtaining informed consent?	82 (81.19)	20 (18.81)
Do you obtain informed consent from the patient for the use of sedation or anesthesia during the dental procedure?	93 (92.08)	9 (7.92)

In the bivariate analysis, the male respondents were significantly associated with a high level of knowledge of the informed consent process compared with their female counterparts (*P =* 0.050). The older Age group of the respondents was significantly associated with a high level of knowledge of the informed consent process compared to the younger counterparts (*P =* 0.016). The years of study and training about informed consent in dental care were not significantly associated with the level of knowledge of the informed consent process (Table 5).

**Table 5:** Association between socio-demographic characteristics and the level of knowledge of informed consent for oral care among the respondents

Variable	Knowledge Level		Chi-Square (df)	*P*-value
	High *n* (%)	Low *n* (%)		
**Sex**				
Male Female	67 (100) 33 (94.3)	0 (0.00) 2 (5.7)	3.85 (1)	0.050
**Age**				
20–29 years 30 years and above	84 (100) 14 (92.3)	0 (0.00) 4 (7.7)	5.81 (1)	**0.016**
**Year of study**				
3rd 4th 5th	25 (100) 30 (100) 45 (95.7)	0 (0.00) 0 (0.00) 2 (4.3)	2.34 (3)	0.504
**Have you ever received any training on the informed consent process in dental care?**				
Yes No	7 75 (98.7) 24 (96.0)	1 (1.3) 2 (4.0)	0.69 (1)	0.403

The year of study training on the informed consent process in dental care was significantly associated with the attitude of students towards the informed consent process (*P* < 0.05, Table 6).

**Table 6:** Association of socio-demographic characteristics and attitudes about informed consent for oral care among the respondents

Variable characteristics	Attitude		Chi-Square (df)	*P*-value
	Positive *n* (%)	Negative *n* (%)		
**Sex**				
Male Female	63 (95.5) 31 (88.6)	4 (4.5) 4 (11.4)	1.6799 (1)	0.195
**Age**				
20–29 years 30 above	56 (75.7) 22 (84.6)	18 (24.3) 4 (15.4)	0.896 (1)	0.344
**Year of Study**				
3rd 4th 5th	13 (52.0) 23 (100) 42 (89.4)	12 (48.0) 7 (17.9) 5 (10.6)	14.04 (2)	0.003
**Have you ever received any training on the informed consent process in dental care?**				
Yes No	7 63 (82.9) 16 (64.0)	13 (17.1) 10 (36.0)	3.941 (1)	**0.047**

There were no socio-demographic factors that were significantly associated with clinical practices of the informed consent process for oral care (*P* > 0.05, Table 7).

**Table 7:** Association between socio-demographic characteristics and clinical practices on informed consent process for oral care among respondents

Variable characteristics	Clinical practices		Chi-Square (df)	*P*-value
	Often *n* (%)	Less often *n* (%)		
**Sex**				
Male Female	57 (86.4) 31 (88.6)	10 (13.6) 4 (11.4)	0.099 (1)	0.75
**Age**				
20–29 years 30 above	67 (86.5) 23 (88.6)	10 (13.5) 5 (11.4)	0.066 (1)	0.79
**Year of Study**				
3rd 4th 5th	20 (80.0) 23 (82.1) 44 (93.6)	5 (20.0) 6 (17.9) 3 (6.4)	3.66 (3)	0.30
**Have you ever received any training on the informed consent process in dental care?**				
Yes No	7 75 (98.7) 24 (96.0)	1 (1.3) 2 (4.0)	0.69 (1)	0.403

When multivariate logistic regression analyses were used to determine the risk factors, there was no significant socio-demographic factor associated with the level of knowledge, attitude, and clinical practices of the informed consent process among the respondents (*P* > 0.05).

**Table 8:** Adjusted Odds ratios of factors associated with knowledge level of informed consent for oral healthcare among respondents

Variable characteristics	Knowledge Level		Adjusted OR (95% CI)
	High *n* (%)	Low *n* (%)	
**Sex of the respondent**			
Male Female	67 (100) 33 (94.3)	0 (0.00) 2 (5.7)	1.0 0.09 (0.393–0.429)
**Age of respondent**			
20–29 years 30 above	84 (100) 14 (92.3)	0 (0.00) 4 (7.7)	1.0 0.889 (0.434–0.500)
**Year of Study**			
3rd 4th 5th	25 (100) 30 (100) 45 (95.7)	0 (0.00) 0 (0.00) 2 (4.3)	1.0 0.982 (1.979–2.026 0.992 (2.005–3.189)
**Have you ever received any training on the informed consent process in dental care?**			
NO YES	24 (96.0) 75 (98.7)	2 (4.0) 1 (1.3)	1.0 0.877 (0.476–0.558)

There were no variables that were significantly associated with the Knowledge level of the informed consent process for oral healthcare after performing the multivariate logistic regression and adjusting for all the other factors (Table 8).

**Table 9:** Adjusted odds ratios of factors associated with attitude about informed consent for oral healthcare among respondents

Variable characteristics	Attitude		Adjusted OR (95% CI)
	Positive *n* (%)	Negative *n* (%)	
**Sex of the respondent**			
Male Female	63 (95.5) 31 (88.6)	4 (4.5) 4 (11.4)	1.0 0.361 (1.170–2.468)
**Age of respondent**			
20–29 years 30 above	56 (75.7) 22 (84.6)	18 (24.3) 4 (15.4)	1.0 0.951 (0.371–0.485)
**Year of Study**			
3rd 4th 5th	13 (52.0) 23 (100) 42 (89.4)	12 (48.0) 7 (17.9) 5 (10.6)	1.0 0.542 (1.366–2.603) 0.514 (1.319–2.638)
**Have you ever received any training on the informed consent process in dental care?**			
YES NO	7 63 (82.9) 16 (64.0)	13 (17.1) 10 (36.0)	1.0 0.987 (0.410–0.431)

There were no variables that were significantly associated with an attitude about informed consent for oral healthcare after performing the multivariate logistic regression and adjusting for all the other factors (Table 9).

## Discussion

This study assessed knowledge, attitude, and practice of the informed consent process in oral healthcare among dental students at Makerere University Dental Hospital.

Knowledge of the informed consent process was generally very high (above 90%) among the respondents which supports a study conducted in Pakistan among dentists where 80% had good knowledge of the informed consent, 17% had fair knowledge and 3% had poor knowledge [5]. Additionally, in a study among postgraduate students and graduate interns in the RV Dental College Hospital in Bengaluru, India, 98.5% of the participants were aware of the informed consent process, 92% of the participants obtained informed consent while 71% obtained written form [2]. This very high percentage of knowledge about the informed consent process among the respondents can be due to increased consciousness of patients’ dentists’ relationship, advancement in dental treatment and quality and the medico-legal issues coming within the practice and the training in both law and medical ethics aim at safeguarding a good standard of medical practice within the community [2, 5].

The age of the respondents was statistically significantly associated with the knowledge level of the informed consent process as dental students who were 30 years and older had a higher level of knowledge. However, at the multivariate level, after adjusting for all the other factors, none of the variables was significantly associated with the knowledge level of the informed consent process for oral healthcare. This finding is in line with a study conducted in Italy about factors associated with nurses’ opinions and practices regarding information and consent [17]. This may be explained that, as age increases, so does work experience, increasing exposure to training and shared experience. As a result, their knowledge about the informed consent process increases. However, it is not in line with the study conducted in Pakistan regarding informed consent where 76.4% of the dentists were younger and aged 23–30 years [5]. This could be because they received more education during their training as compared to their older colleagues [5], which was not the case in this study. It was also observed in this study that dental students scored high (98.0%) on the statement “It is the patient’s right to be informed about the treatment”. Being aware that patients have rights in healthcare is important as it ensures that patients are treated with respect and dignity. Patients are entitled to their point of view and they can also choose to accept or refuse treatment as stipulated in the Uganda Patient’s Rights and Responsibilities Charter [18].

Furthermore, most of the students (91.2%) were aware that it is a legal requirement to obtain informed consent. In dental practice, students need to have full knowledge of the informed consent process because they must give patients information about a given procedure including the risks and benefits. If patients are not provided with information regarding the risks of a procedure, especially an invasive procedure and they get injured or experience an adverse effect, it can lead to litigation which can be costly for dentists in terms of time, resources, and loss of clients. Dentists’ reputation is also compromised when they are involved in lawsuits. In this era, the number of lawsuits is rising, hence when dentists are aware that it is a legal obligation to obtain consent, this helps them to be cautious and keen on giving patients all the necessary information to ensure an informed consent is obtained. Most students (92.2%) also knew that informed consent was not only required for complex procedures. Even for simple procedures, patients need to make a decision regarding treatment after they are properly informed, hence, simple procedures should not be taken for granted. For 81.4% of the respondents, they knew that informed consent is an ongoing process and not a one-off. This is important because dental professionals must keep communicating with their patients. Some treatments are done through different phases or steps that require multiple visits, for instance when making complete dentures. Dentists must keep giving information and answering patients’ questions at each step to ensure the success of a treatment. Most participants were aware that a patient cannot consent if they are under the influence of medication or alcohol. This is an important consideration because patients must be attentive so that they can process and understand the information they are given [19, 20]. If they are under the influence of alcohol or drugs, which impair judgment, they cannot provide valid voluntary consent.

Most dental students had a positive attitude towards the informed consent process and this was similar to studies conducted in India by Veeresh et al. [7] and Vandana et al. [21] which indicated that 96% of dental professionals recognized the importance of informed consent in dental care. The positive attitude was due to the awareness of the code of dental ethics which states that any treatment procedure without patient informed consent will breach the ethical principles, high level of training, and more work experience. Moreover, the dentists are encouraged to provide a copy of the consent document to the patient so that this copy can be revisited by the patient and read out of the dental office [7, 22]. Students with positive attitudes toward the informed consent process were more likely to practice adequate informed consent than those with negative attitudes. This finding is comparable with studies [5, 23] in Pakistan in auditing the knowledge and attitudes of doctors towards informed consent in surgical procedures. This could be due to the increased awareness of the benefits of obtaining informed consent and also its inclusion in the academic curriculum during their three years of biomedical sciences. A positive attitude towards informed consent practice is fundamental and enhances motivation for practice.

Having received training about informed consent and the year of study were statistically significantly associated with a positive attitude towards the informed consent process. Students who had received training were more likely to have a higher positive attitude towards the informed consent process than those who had not received training. However, on further analysis and after adjusting for all other factors, none of the variables was significantly associated with attitude regarding the informed consent process for oral healthcare. This is in line with a study by Negash et al. [24] about practice and factors associated with the informed consent process for major surgical procedures among healthcare workers in South-Eastern Ethiopia. This is because training can update, boost, clarify, and advance the attitudes of health workers as it comprises the components of informed consent, patient autonomy, and patient-centered care.

Most of the respondents agreed that informed consent was important in dental care, an important attitude that can influence the behaviours of their colleagues [9]. The informed consent process is intended to respect patient autonomy by ensuring that they decide on a proposed treatment [25]. Today, patient-centered approaches to decision-making are highly considered. With advancements in technology and increased access to the internet, smartphones, and laptops, people are learning more about health issues and their rights and are increasingly getting interested in asking questions about any treatment they are going to receive [26].

Less than a third of the respondents felt that informed consent leads to better patient outcomes and satisfaction while less than a quarter felt that the informed consent process builds trust between the dentist and the patient. This supports the belief that the informed consent process is essential in the provider-patient relationship, shared decision-making instead of the paternalistic approach to patient care where the health practitioner knows it all, and respects to patient’s autonomy by ensuring that they decide on a proposed treatment (patient-centered approaches to decision making) leading to patients’ compliance or adherence hence patient outcomes and satisfaction [25–28]. However, in this present study majority of the respondents had a negative views about the informed consent leading to better patient outcomes, trust and satisfaction. This could be attributed to the fact that they have limited years of work experience and they are yet to realize that informed consent is not only about accepting treatment, it is an interactive process which involves both parties sharing opinions and learning from each other to come up with an effective treatment plans.

The respondents’ clinical practices regarding the informed consent process were generally high (above 85%) which supports a study conducted in Karachi among dental students and graduates where 88.8% practiced informed consent [29]. Additionally, in a study among postgraduate students and graduate interns in the RV Dental College Hospital in Bengaluru, India, 92% of the participants obtained informed consent, of which 71% obtained written consent [2].

In this present study, most of the respondents explained the patient’s condition and procedures for treatment, in line with recommendations from other authors [13]. This makes it easier for patients to understand, and appreciate the treatment and how it is going to help them solve a problem and improve their quality of life. Most respondents obtained written consent from patients, in contrast to previous studies [5, 10]. When dentists obtain written informed consent, this offers them some form of protection in case a patient raises any complaint.

### Study strengths and limitations

This study was the first of its kind among dental students, thus providing baseline data. The study population was based on census recruitment, which ruled out sampling error. A self-administered questionnaire was used in the survey, enabling the anonymity of the respondents and eliminating interviewer bias. However, self-reporting could have inflated social desirability bias. No observations were made regarding the actual consenting practices of the students. The results from this study may not be generalizable or may be generalized with caution nationwide since the study was conducted in one setting. Also, since the study was cross-sectional, it does not show a cause-effect relationship in the informed consent process.

## Conclusion

Overall, the dental students in Makerere University Dental Hospital had high knowledge, and positive attitudes and most often practiced the informed consent process for oral health care. Also, the year of study, age, and sex of the dental students had a statistically significant association with the informed consent process.

### Recommendation

Continuous training is necessary to remind dental students about the importance of informed consent in healthcare, not only for complex procedures but even for simple treatments. Students need to appreciate the role of consent in the health provider-patient relationship.

### Further research

There is a need for further studies to include dental students from other institutions in the country regarding the informed consent process for better generalization and decision-making. Future studies should include qualitative methods of data collection to provide insightful views and experiences of dental students relating to obtaining informed consent.

## Data Availability

No datasets were generated or analysed during the current study.
